# Correction: Immortalization and characterization of Schwann cell lines derived from NF1-associated cutaneous neurofibromas

**DOI:** 10.1371/journal.pone.0344792

**Published:** 2026-03-10

**Authors:** Hua Li, Alexander Pemov, Robert Allaway, David F. Muir, Lung-Ji Chang, Jineta Banerjee, Alexandra J. Scott, Jaime M.W. Nagy, Jian Liu, Meritxell Carrió, Helena Mazuelas, Anthony Yachnis, Sang Y. Lee, Xiaochun Zhang, Yang Lyu, Douglas R. Stewart, Angela Hirbe, Jaishri O. Blakeley, Eduard Serra, Deeann Wallis, Margaret R. Wallace

The Funding statement for this article is incorrect. The correct Funding statement is as follows: This study has been funded by the Neurofibromatosis Therapeutic Acceleration Program (NTAP)(#185709 awarded to MRW). This publication was supported by a subagreement from the Johns Hopkins University via the NTAP with funds to Dr. Wallace provided by a Grant Agreement from the Bloomberg Family Foundation. This publication’s contents are solely the responsibility of the authors and do not necessarily represent the official views of the Bloomberg Family Foundation or the Johns Hopkins University. This research was also supported in part by the Intramural Research Program of the National Institutes of Health (NIH) (#Z01CP010144-18 awarded to DRS and AP). The contributions of the NIH authors are considered Works of the United States Government. The findings and conclusions presented in this paper are those of the author(s) and do not necessarily reflect the views of the NIH or the U.S. Department of Health and Human Services. Other support for this work includes: A gift from Dan and Jennifer Gilbert (no award number) to the University of Alabama at Birmingham NF program; NTAP cNF initiative project (#185708 awarded to ES); NTAP Data and Knowledge Portal for NF1 (#159101 and #189102 awarded to RA and JB).

## References

[1] Li H, Pemov A, Allaway R, Muir DF, Chang L-J, Banerjee J, et al. Immortalization and characterization of Schwann cell lines derived from NF1-associated cutaneous neurofibromas. PLoS One. 2026;21(1):e0340183. doi: 10.1371/journal.pone.0340183 41564118 PMC12822933

