# Supplementary figures and images for: Immortalization and characterization of Schwann cell lines derived from NF1-associated cutaneous neurofibromas
Source: PLoS One. 2026 Jan 21;21(1):e0340183. doi: 10.1371/journal.pone.0340183 (PMC12822933; doi:10.1371/journal.pone.0340183)

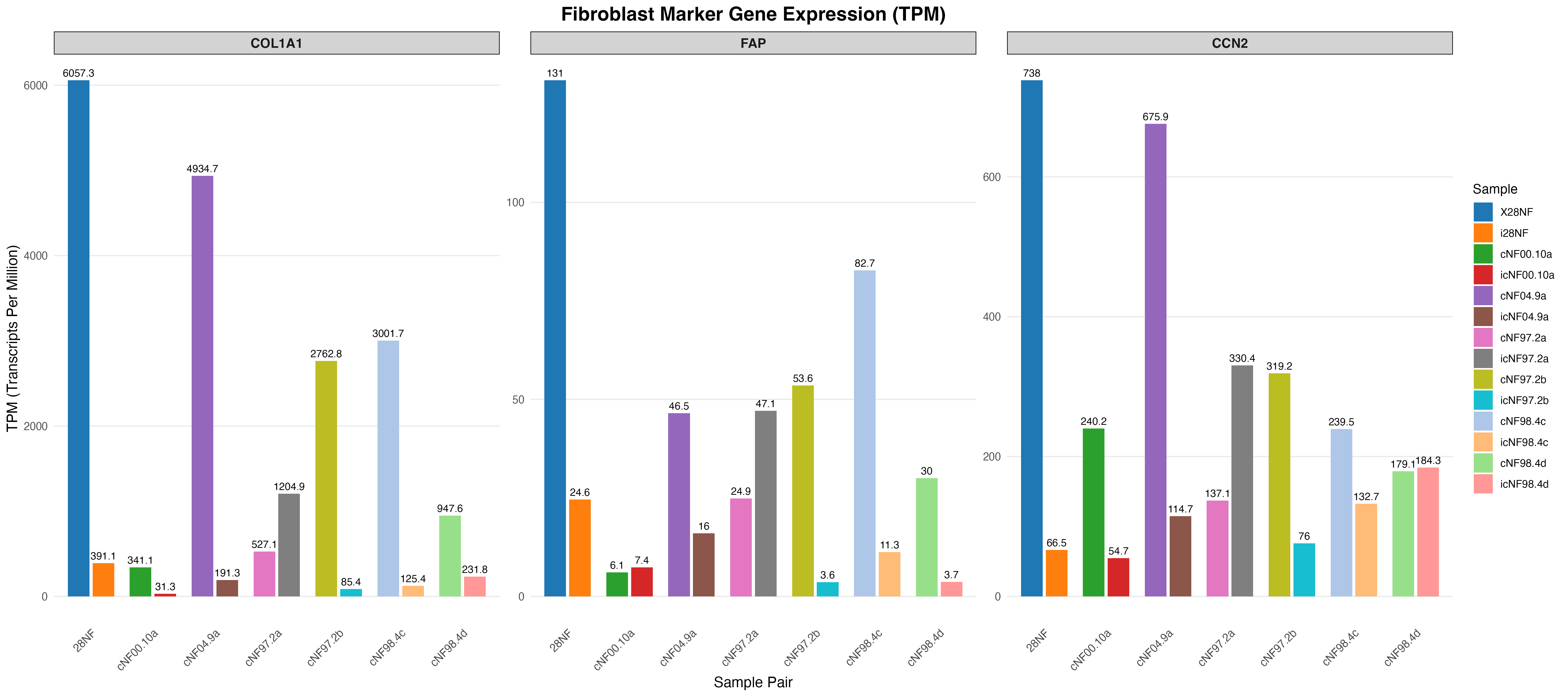

Supplement: S1 Fig — The genes surveyed for fibroblast lineage include COL1A1 (encodes collagen 1A1), FAP (encodes fibroblast activation protein alpha), and CCN2 (encodes connective tissue growth factor). The samples are indicated by different colors per the key, with immortalized:parental cells shown in pairs. Passage number used for each RNA sample used in this and all analyses in this study: 28cNF/i28cNF (p6/p13), cNF00.10a/icNF00.10a (p5/p17), cNF04.9a/icNF04.9a (p5/16), cNF97.2a/icNF97.2a (p4/p14), cNF97.2b/icNF97.2b (p4/p13),cNF98.4c/icNF98.4c (p6/p13), cNF98.4d/icNF97.4d (p5/p15). All but one pair shows reduction in fibroblast transcript count with immortalization. (PNG) [file pone.0340183.s001.png]

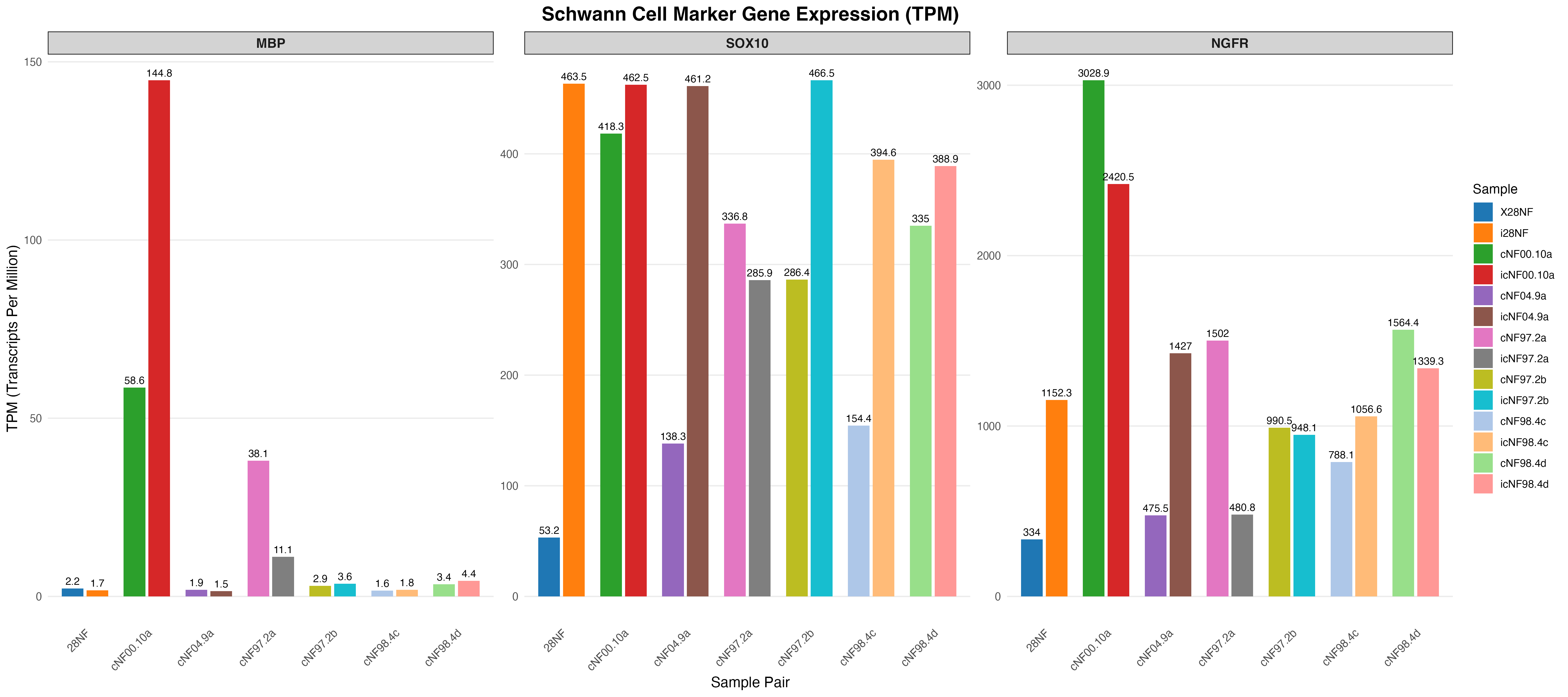

Supplement: S2 Fig — The genes surveyed for Schwann cell lineage include MBP (encodes myelin binding protein), SOX10 (encodes SRY-box transcription factor 10), and NGFR (encodes nerve growth factor receptor). The samples are indicated by different colors per the key, with immortalized:parental cells shown in pairs. All but one pair shows increase in Schwann lineage transcript count with immortalization, consistent with the high percentage of immortalized cells that were positive for S100B staining. (PNG) [file pone.0340183.s002.png]

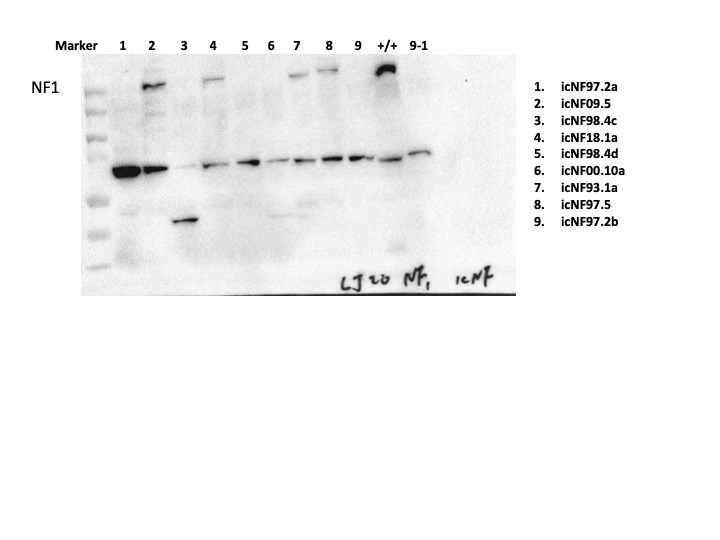

Supplement: Slide 1 — (JPEG) [file pone.0340183.s007.jpeg]

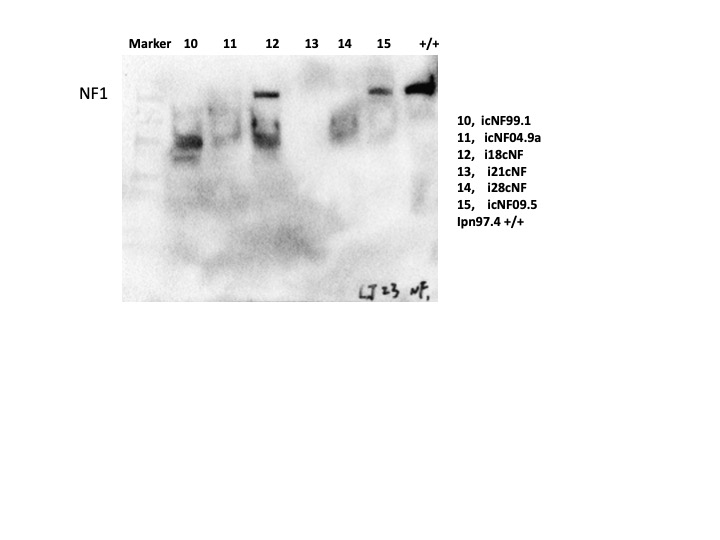

Supplement: Slide 2 — (JPEG) [file pone.0340183.s008.jpeg]

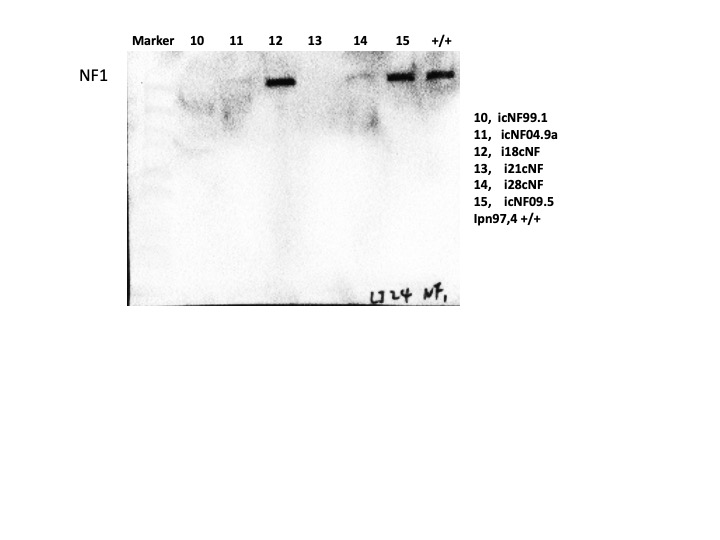

Supplement: Slide 3 — (JPEG) [file pone.0340183.s009.jpeg]

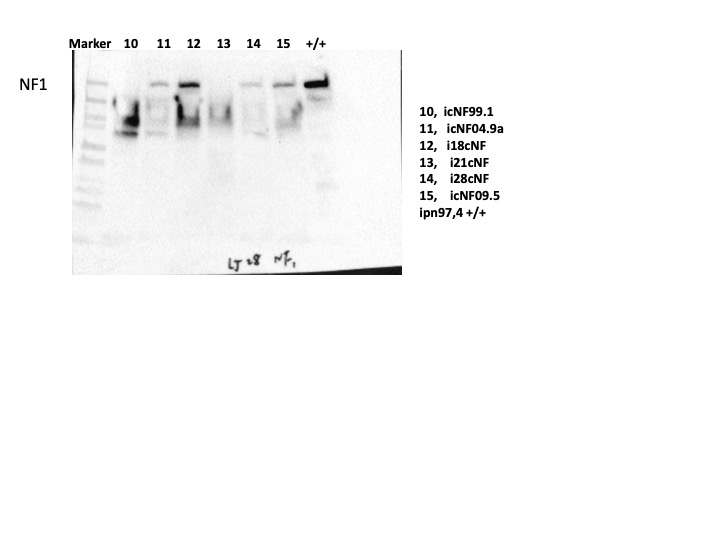

Supplement: Slide 4 — (JPEG) [file pone.0340183.s010.jpeg]

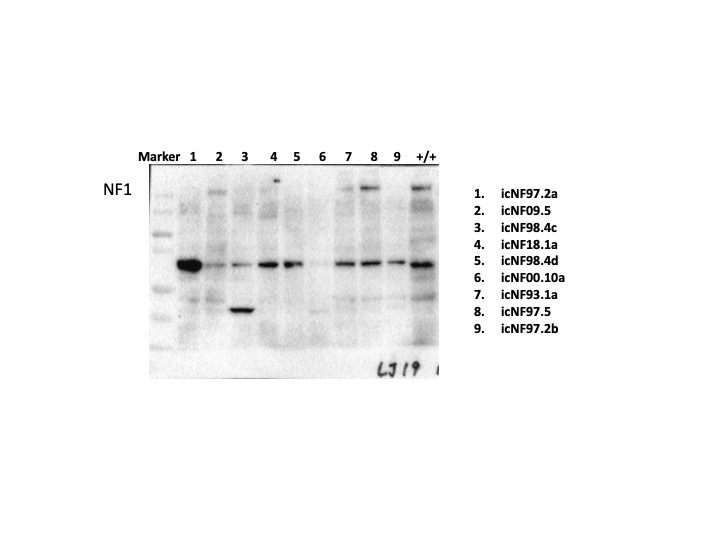

Supplement: Slide 5 — (JPEG) [file pone.0340183.s011.jpeg]
