# Supplementary material for: Immortalization and characterization of Schwann cell lines derived from NF1-associated cutaneous neurofibromas
Source: PLoS One. 2026 Jan 21;21(1):e0340183. doi: 10.1371/journal.pone.0340183 (PMC12822933; doi:10.1371/journal.pone.0340183)
Supplement: S4 Fig — NF1 mutations detected in the primary (top) and immortalized (bottom) cNF cell lines from whole-exome sequencing data. (PDF) [file pone.0340183.s004.pdf]

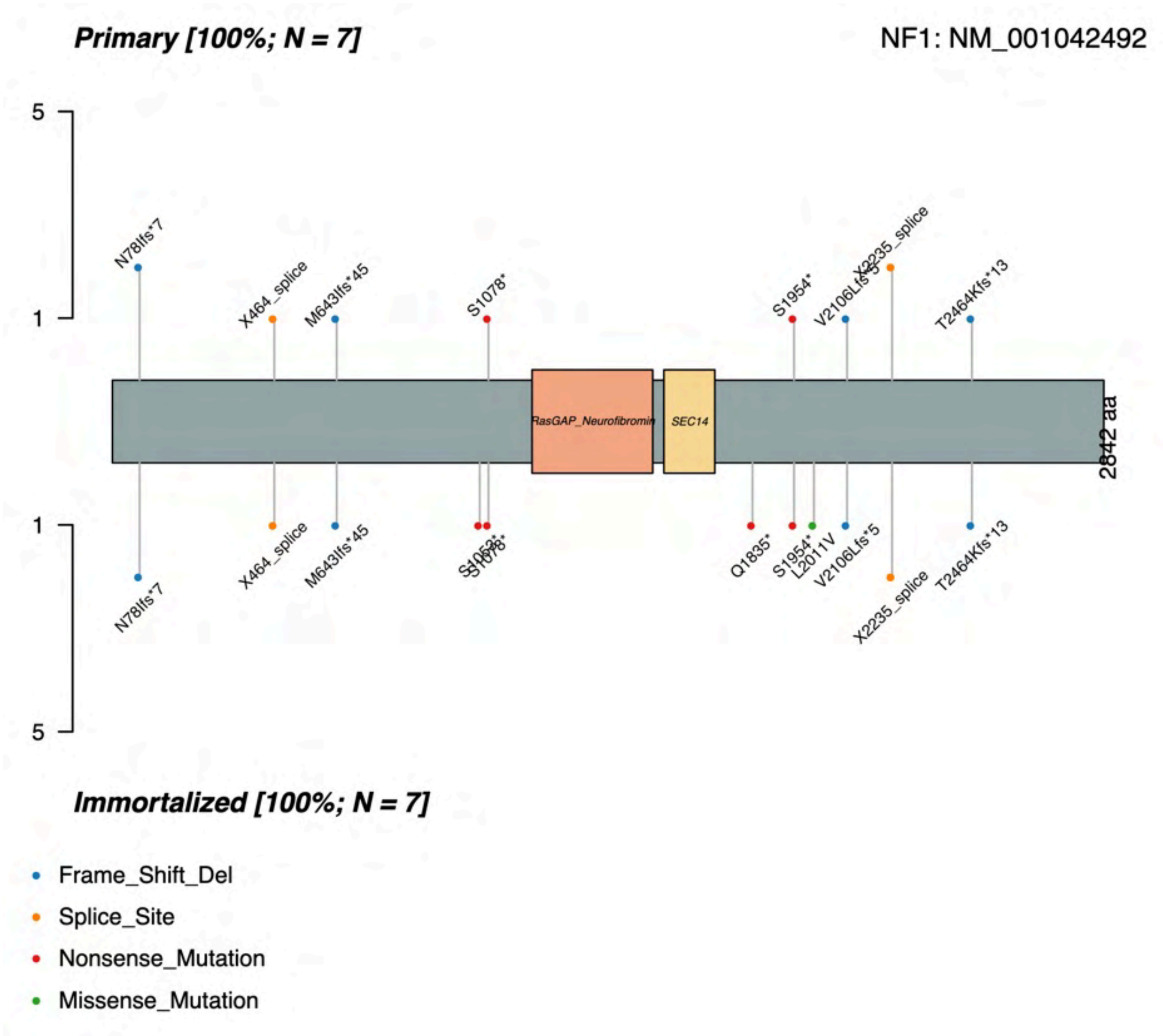

S4 Figure - **NF1 mutations detected in the primary (top) and immortalized (bottom) cNF cell lines from whole-exome sequencing data.**
