# Supplementary material for: Immortalization and characterization of Schwann cell lines derived from NF1-associated cutaneous neurofibromas
Source: PLoS One. 2026 Jan 21;21(1):e0340183. doi: 10.1371/journal.pone.0340183 (PMC12822933; doi:10.1371/journal.pone.0340183)

S5 - S18 Figures (starts next page): **Copy number variation and associated gene expression in all cell lines profiled by whole genome sequencing.** Circos plots show the copy number variation across the profiled genome (red) and the log 10 transformed gene expression at each of those locations. As expected, telomeric and centromeric regions have reduced transcription/ associated gene expression, as do Y chromosome genes in cell lines that have no copies of this chromosome. However, other large "deletions", such as chr4 in cell line 28cNF do not lack transcription of genes in these regions, suggesting that these deletions are likely an artifact. This analysis demonstrates that the sex of each cell line was as expected, and that the immortalization process did result in large copy number alterations in the cNF cell lines.

28cNF

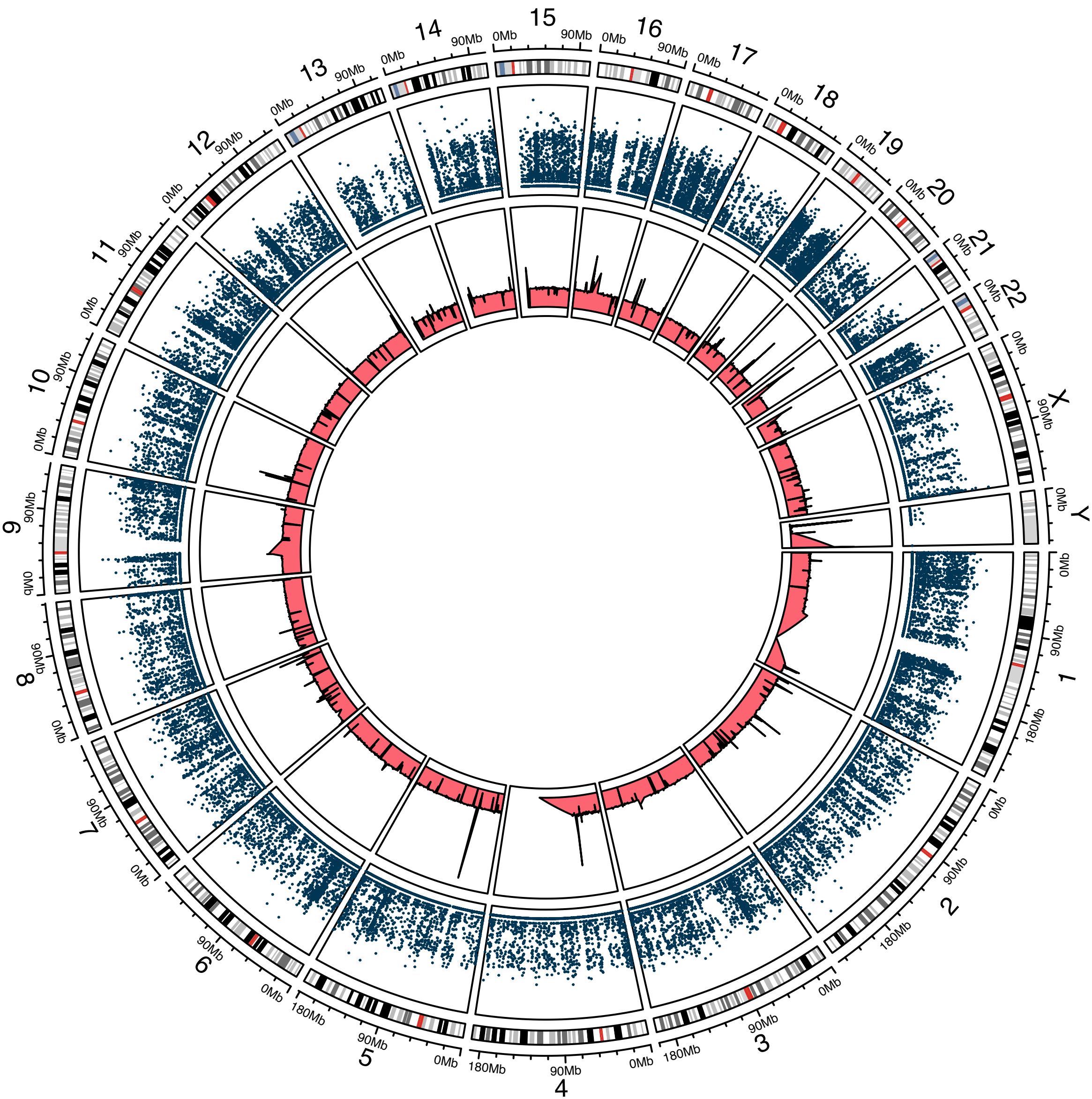

i28cNF

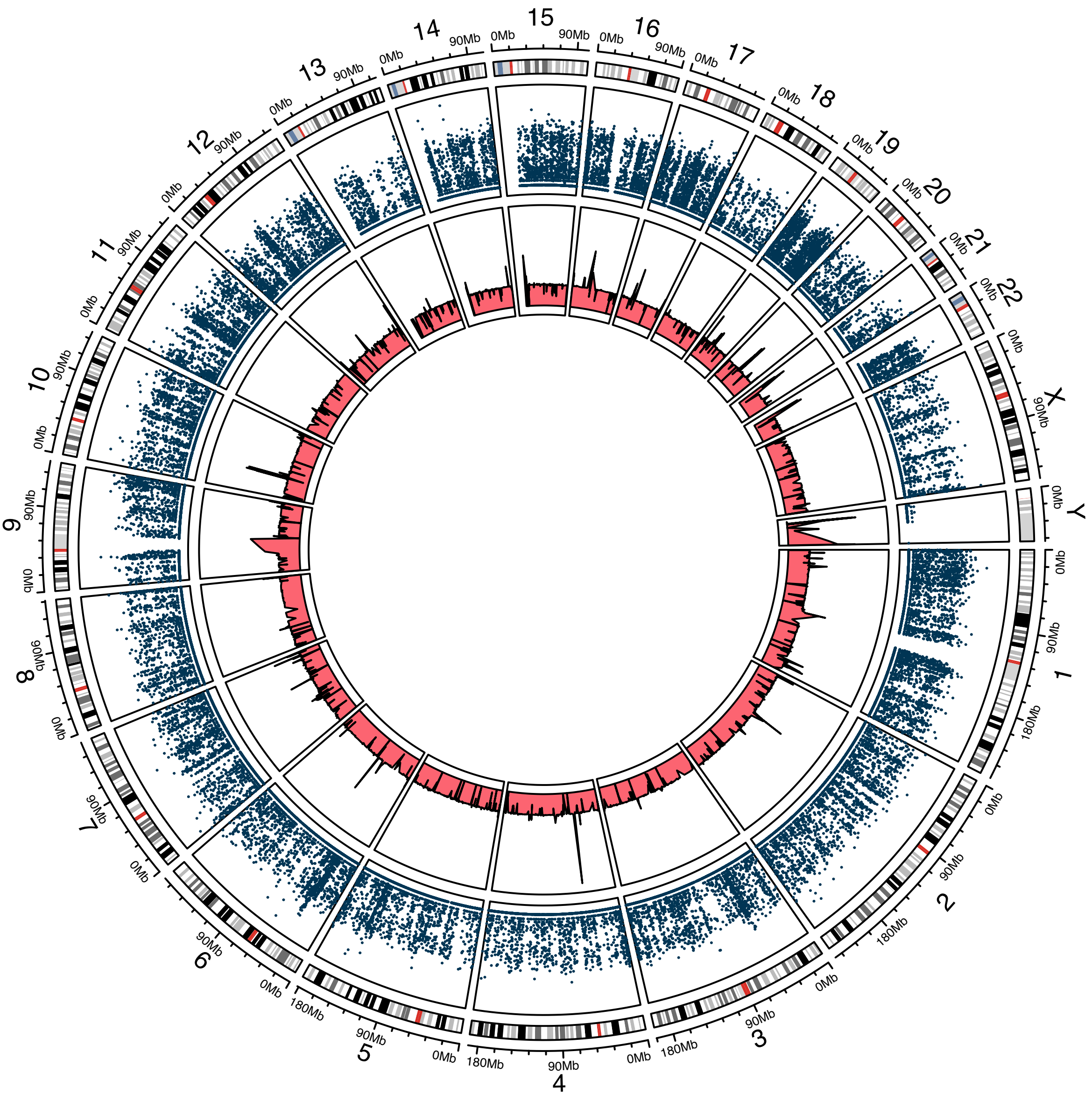

cNF00.10a

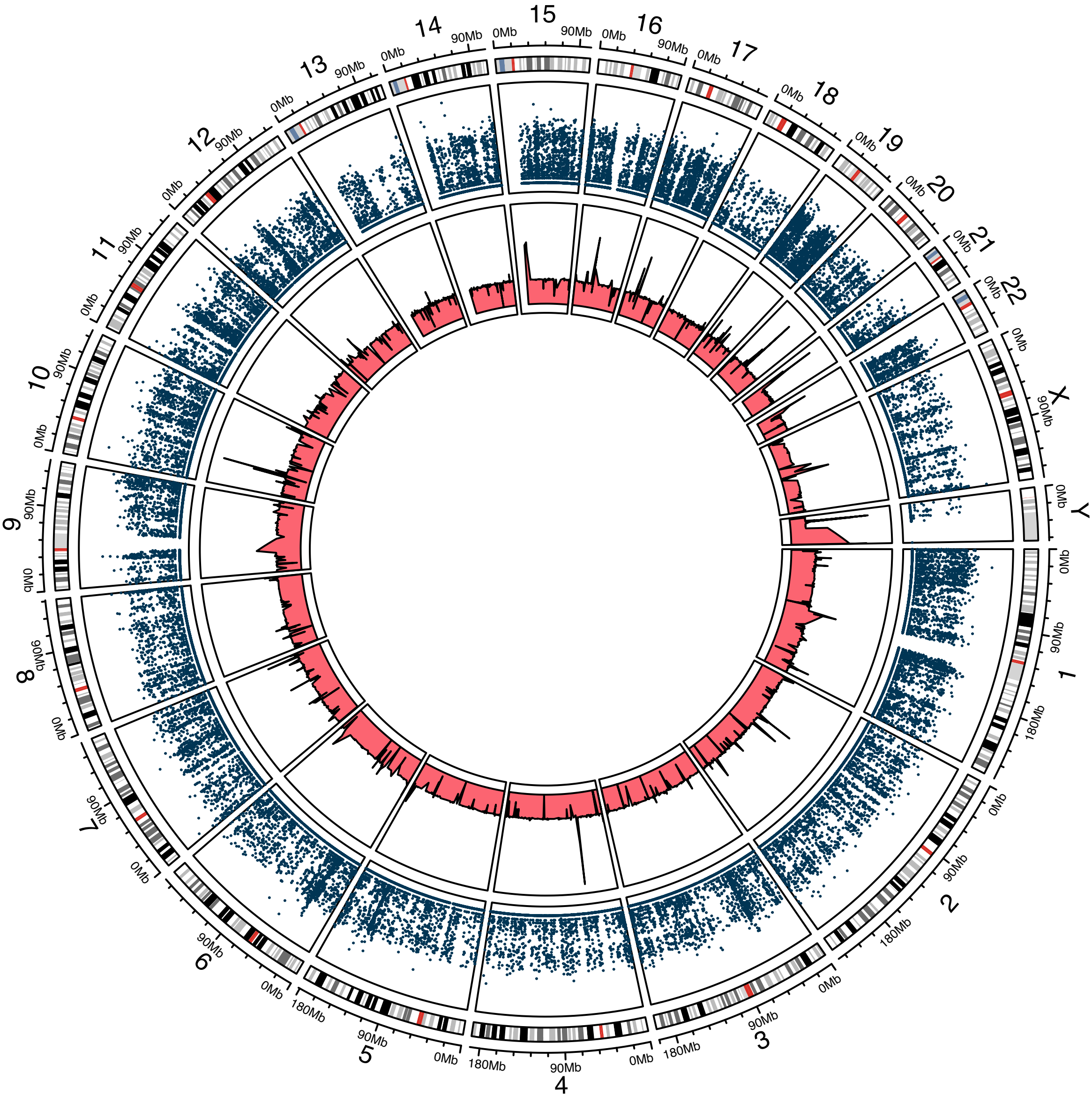

icNF00.10a

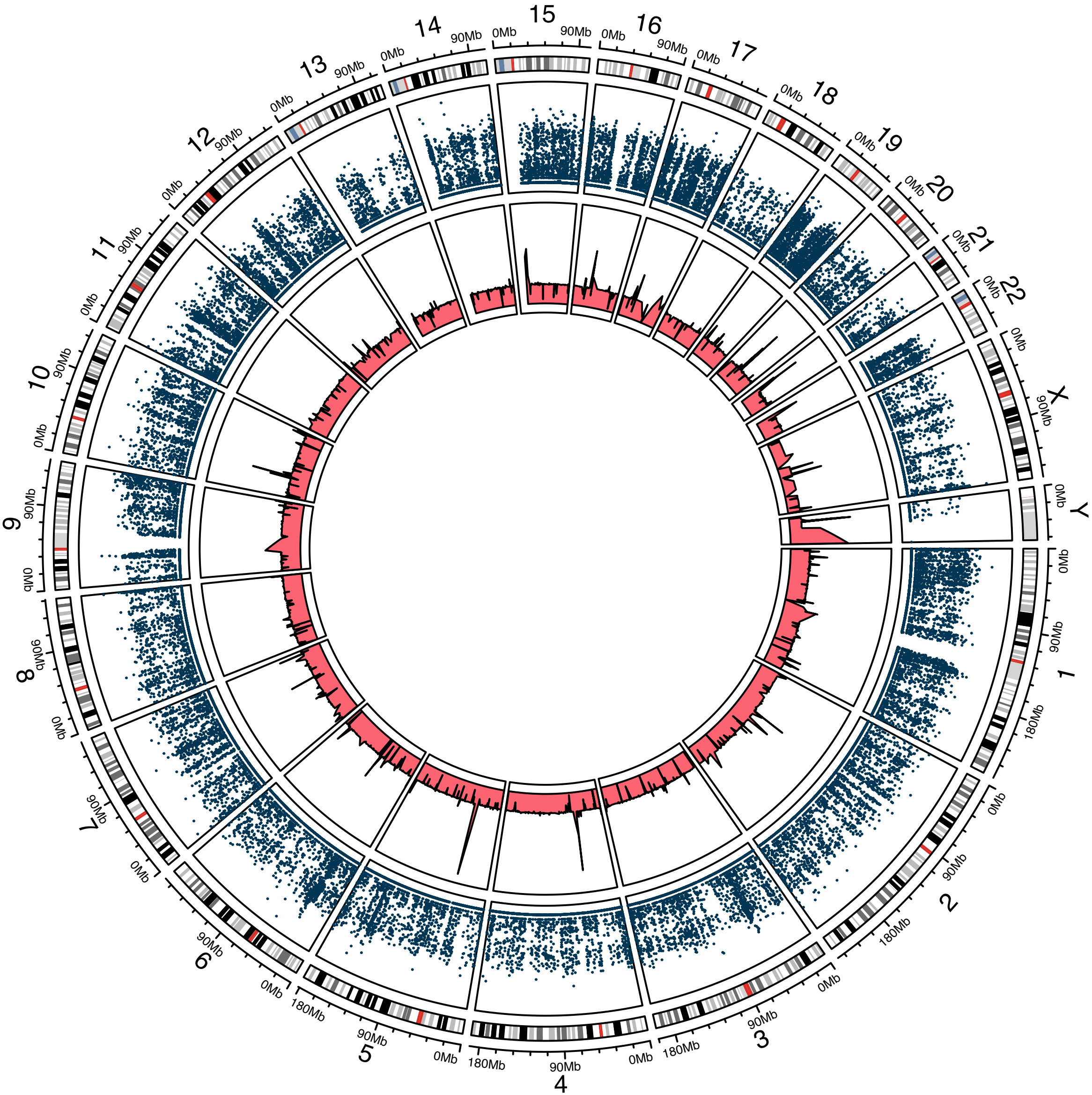

cNF04.9a

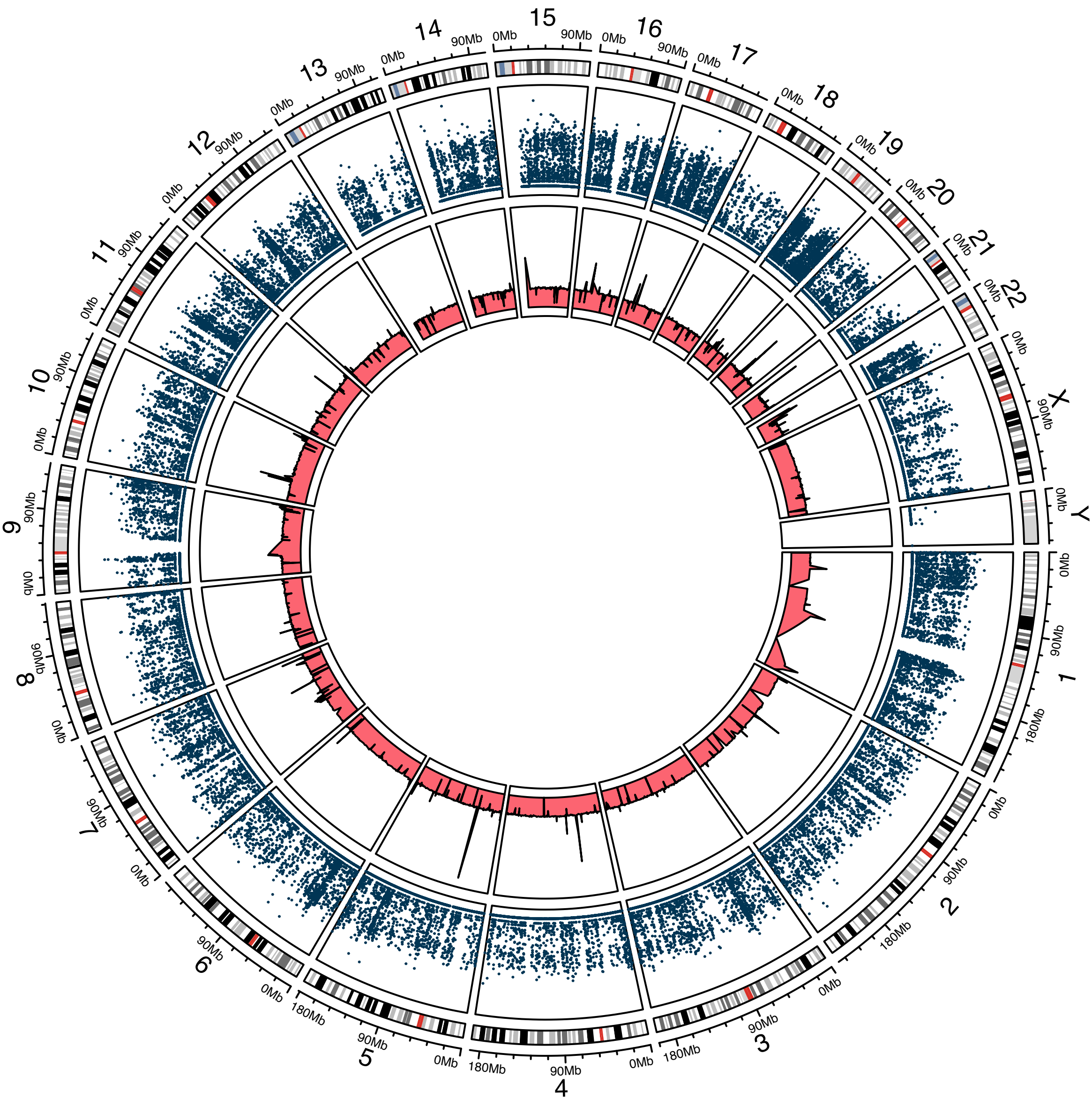

icNF04.9a

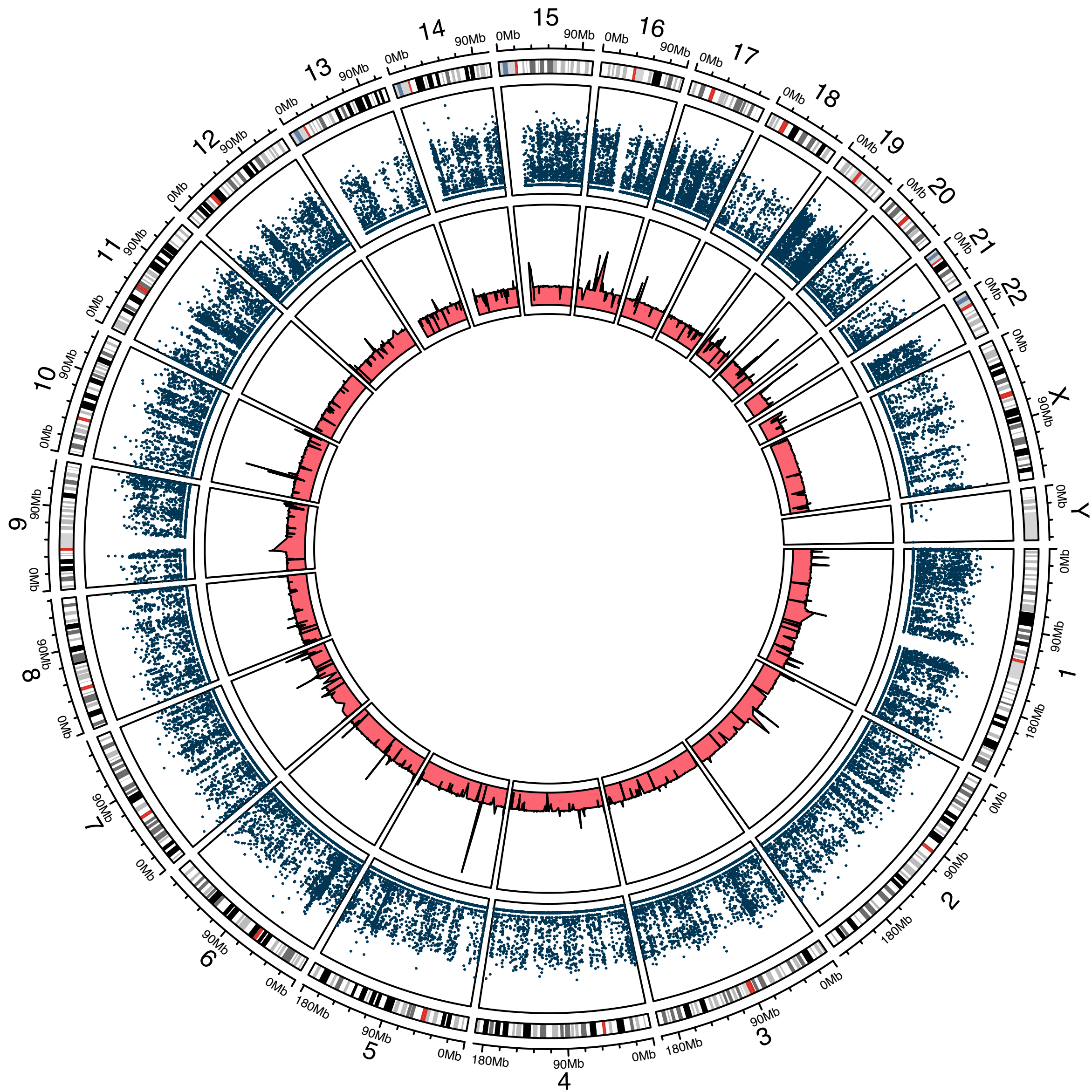

cNF97.2a

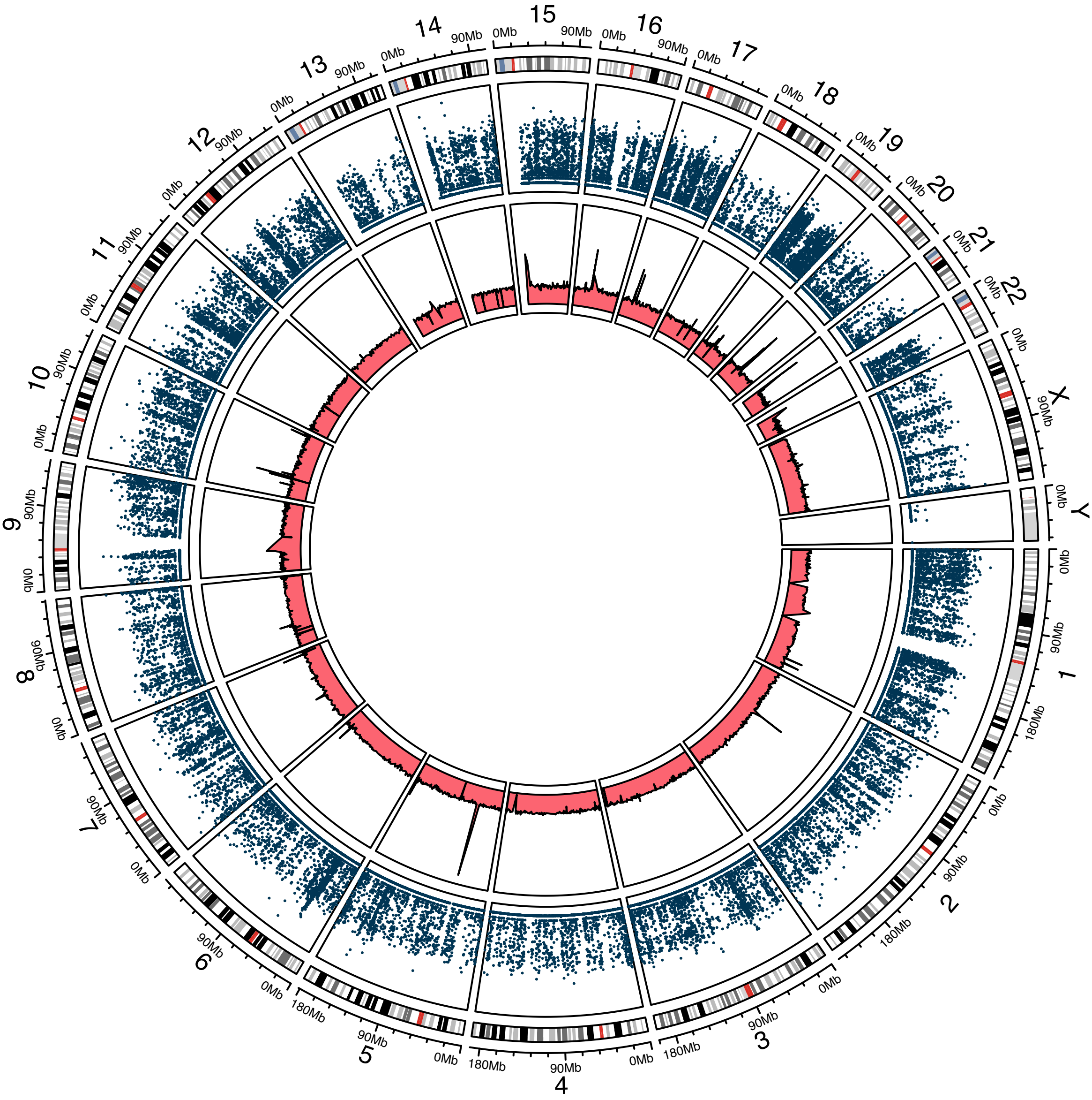

icNF97.2a

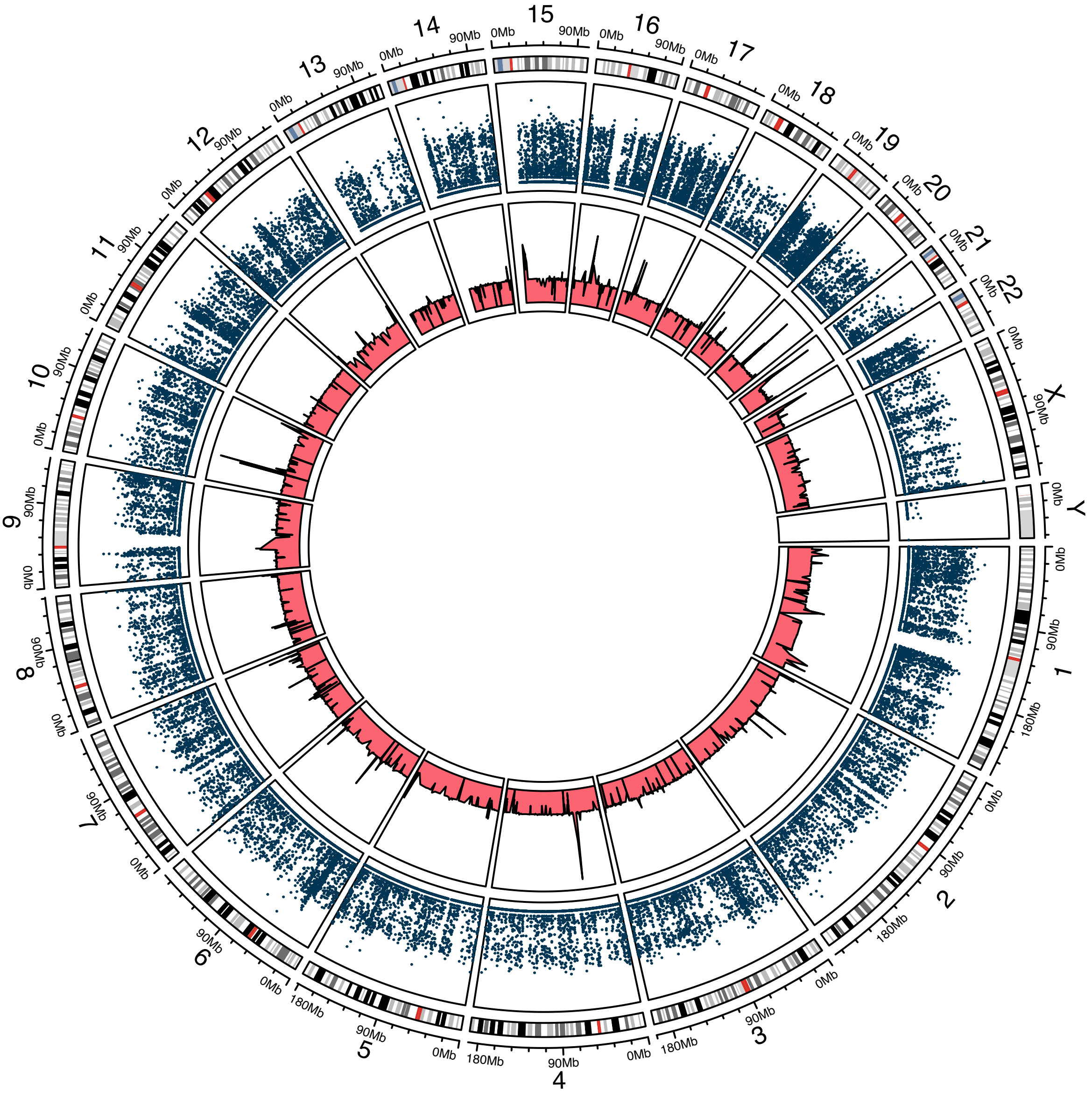

cNF97.2b

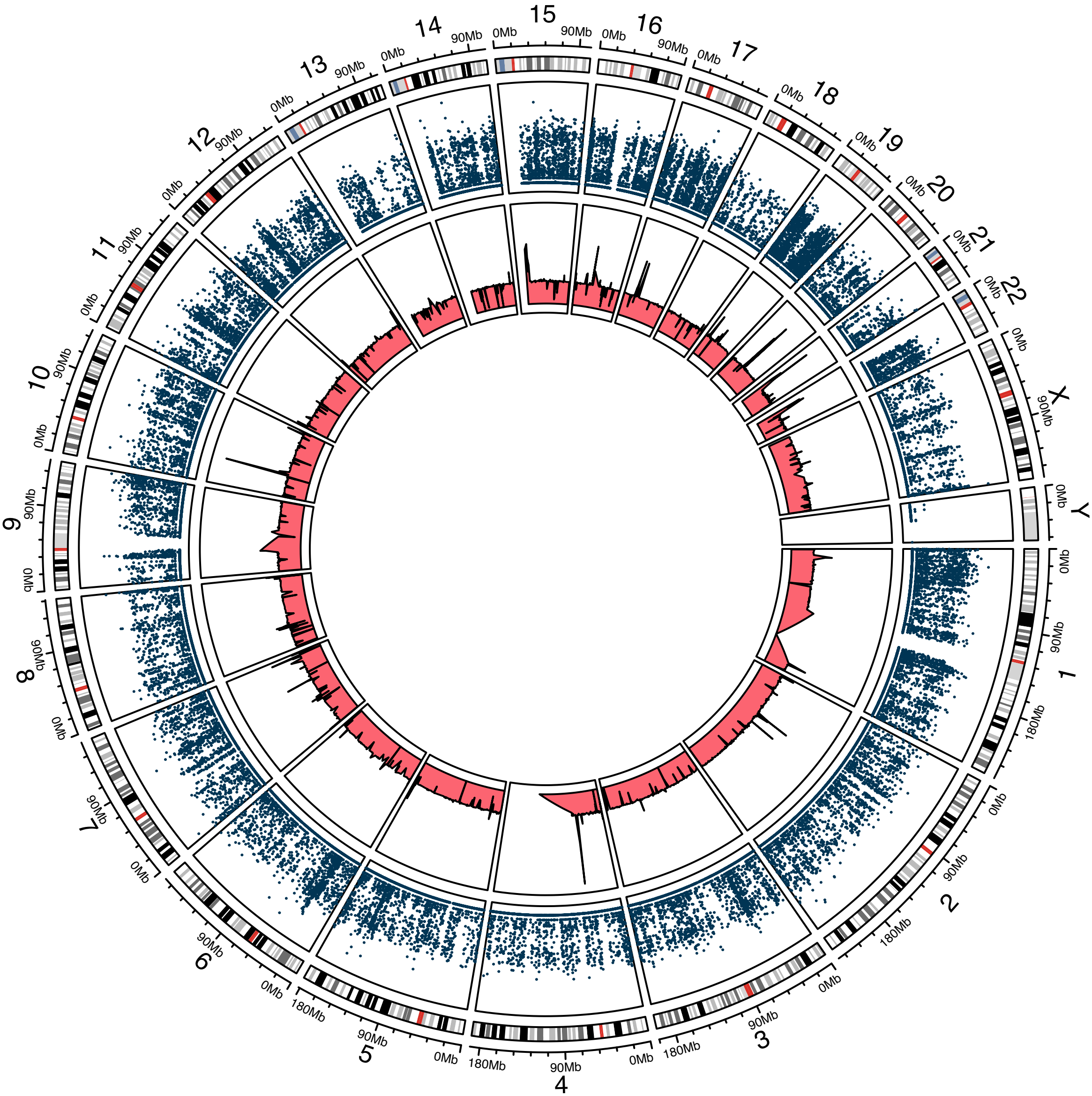

icNF97.2b

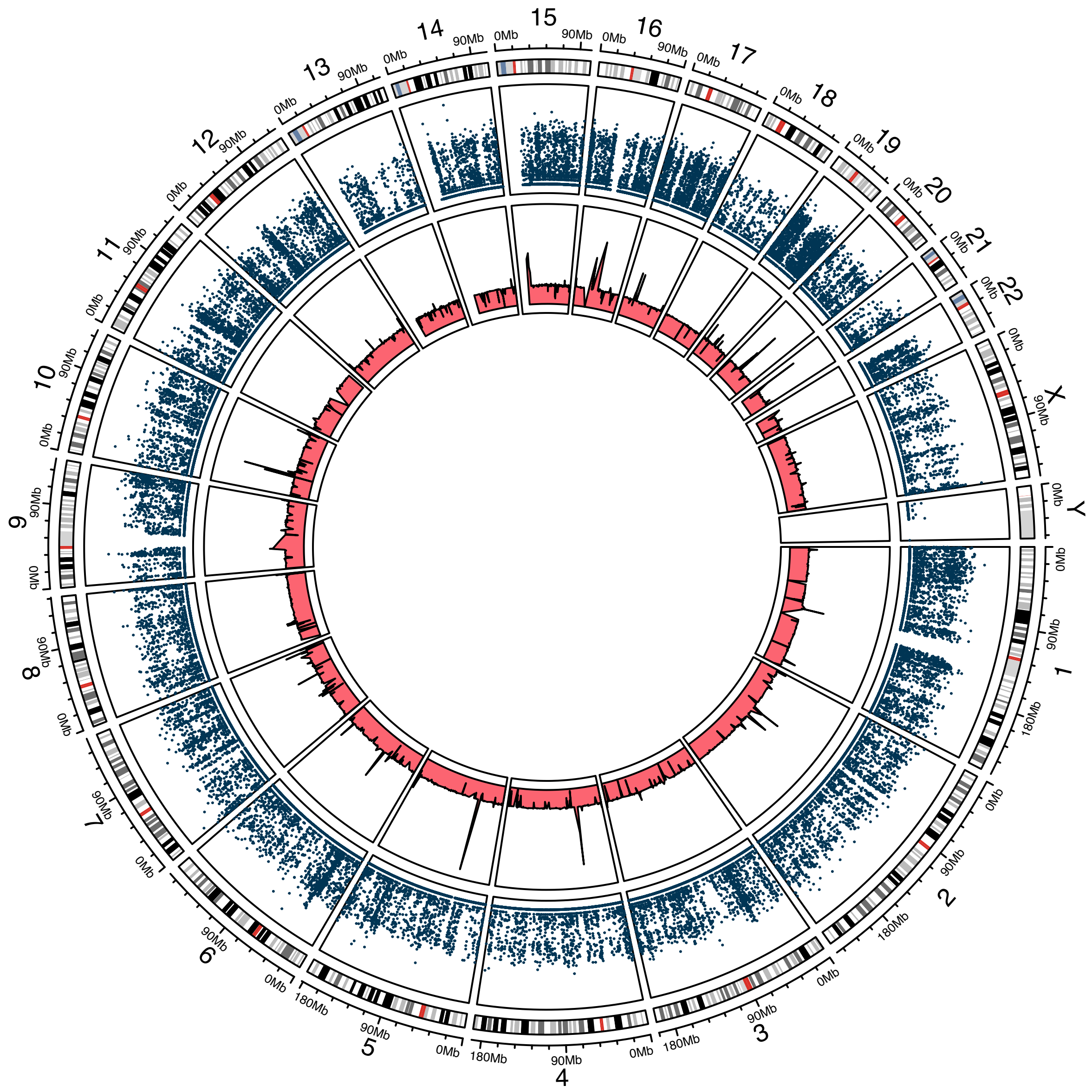

cNF98.4c

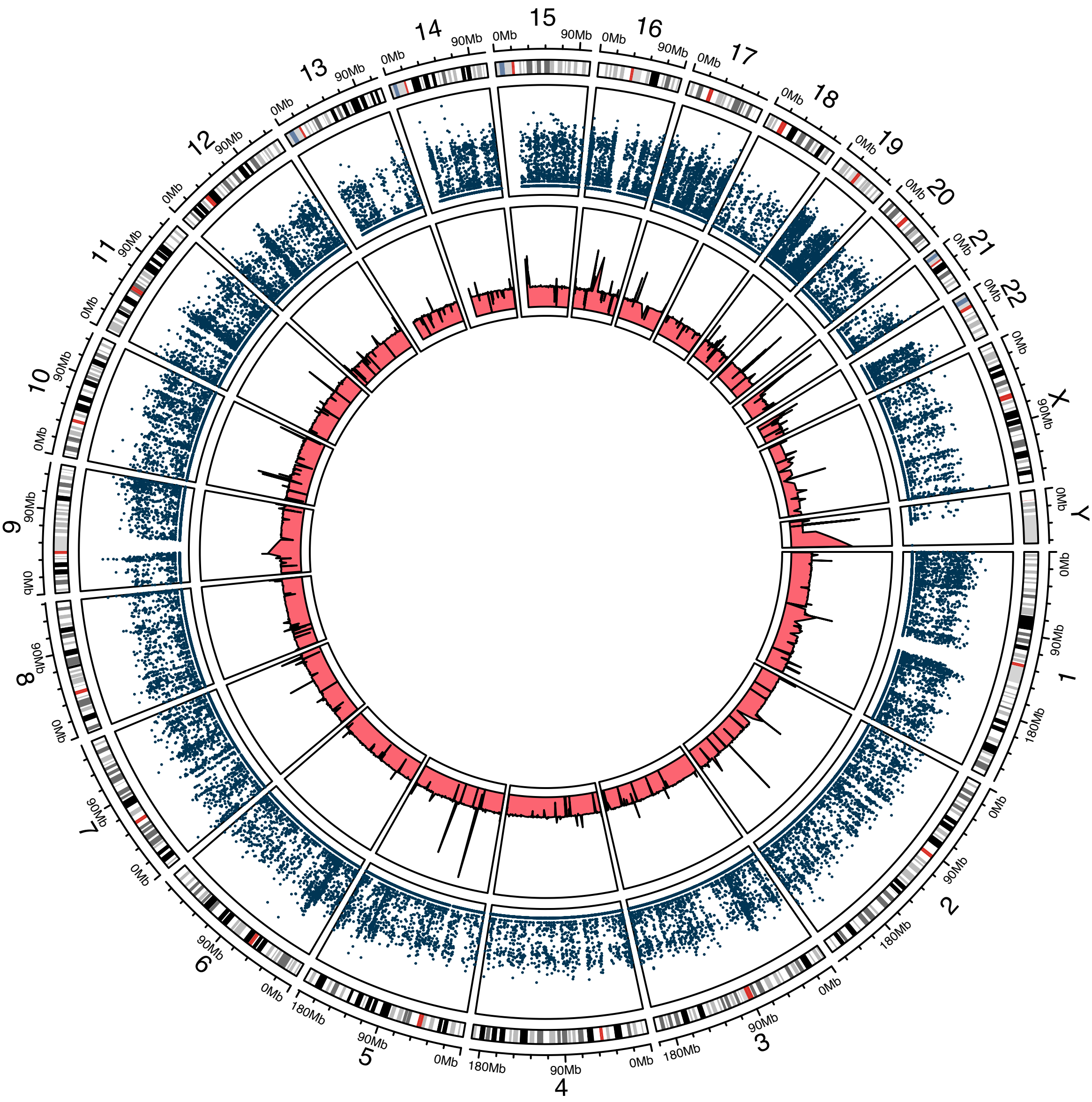

icNF98.4c

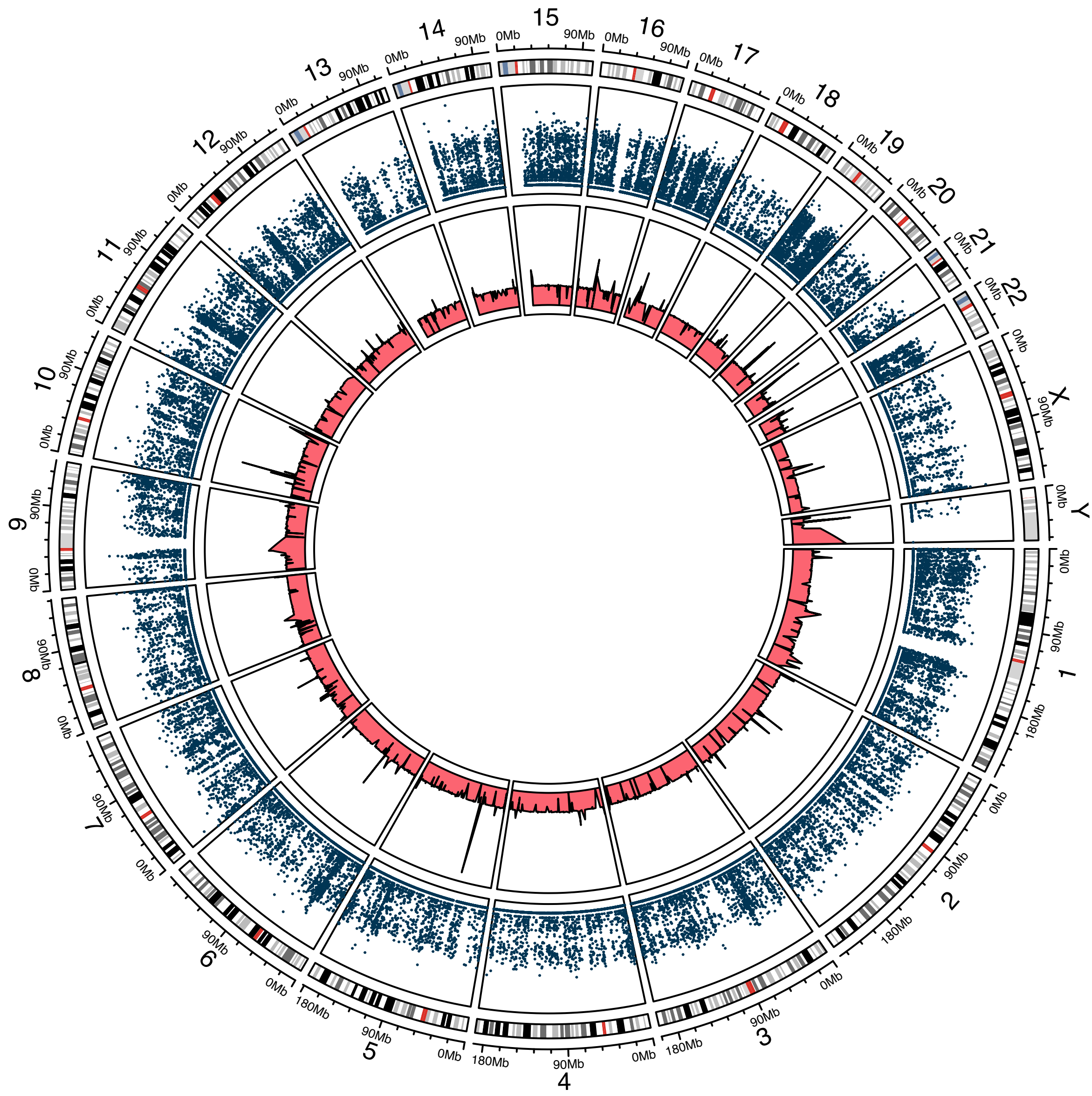

cNF98.4d

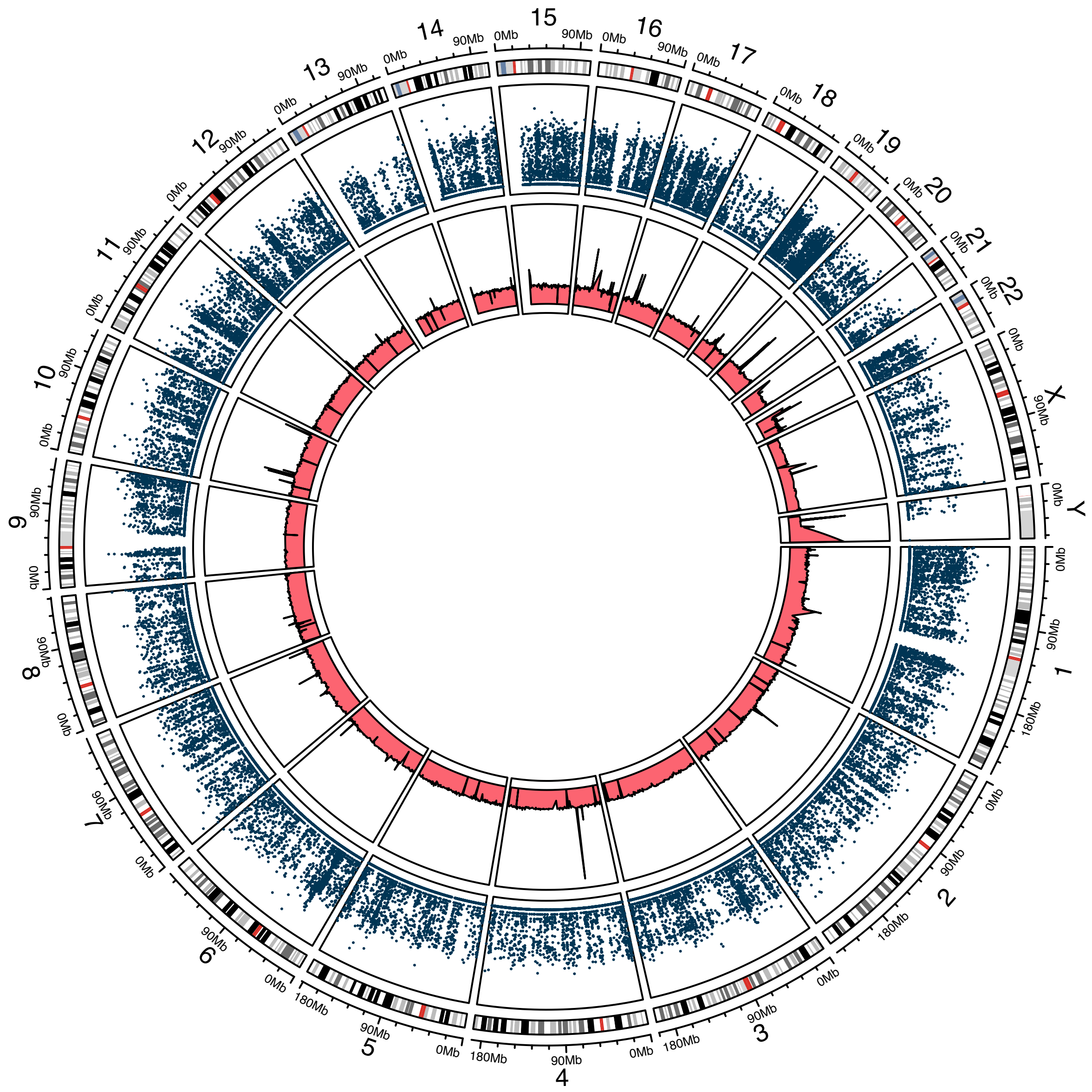

icNF98.4d

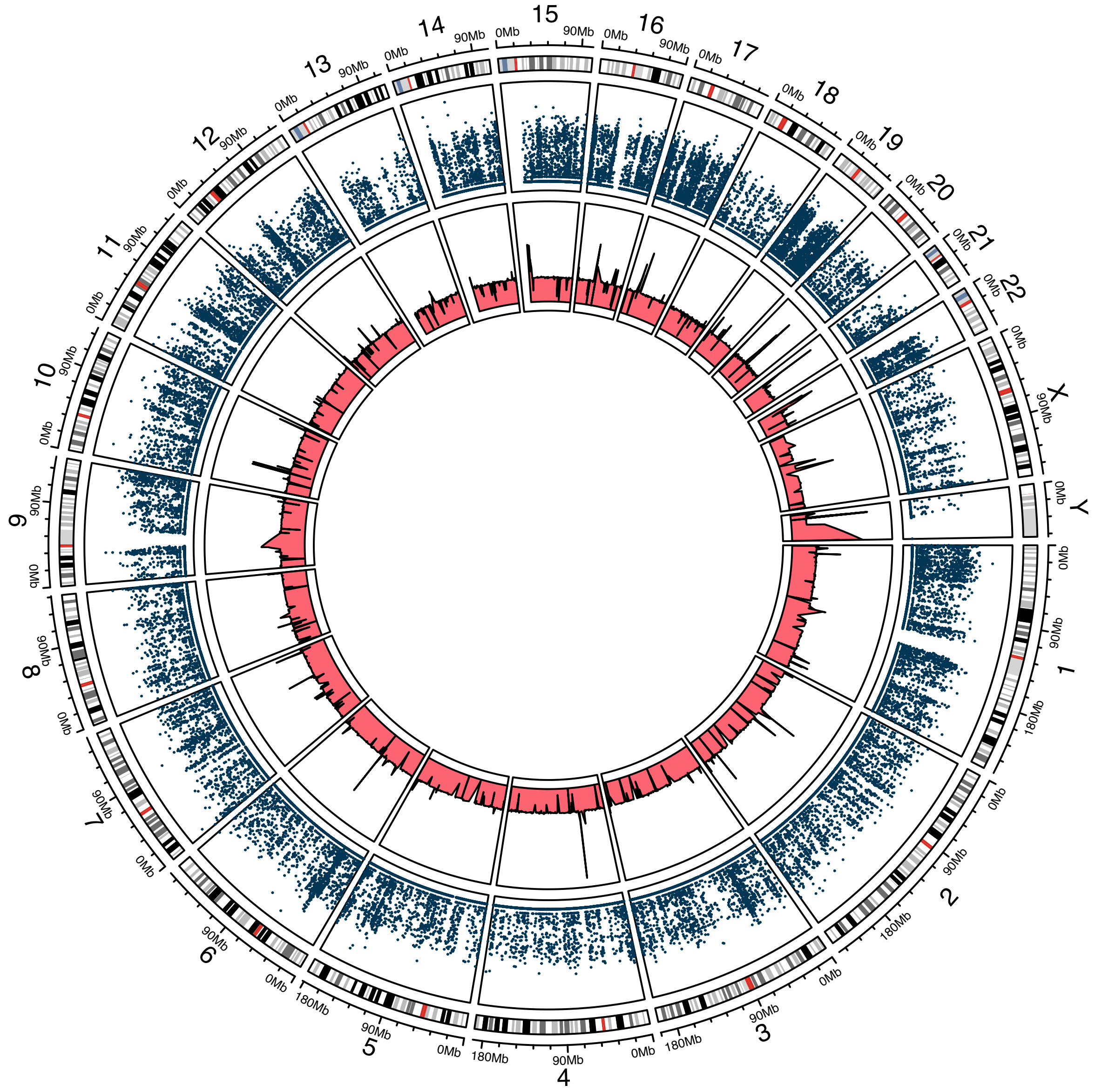

Supplement: S5-18 Fig — Circos plots show the copy number variation across the profiled genome (red) and the log 10 transformed gene expression at each of those locations. As expected, telomeric and centromeric regions have reduced transcription/associated gene expression, as do Y chromosome genes in cell lines that have no copies of this chromosome. However, other large “deletions”, such as chr4 in cell line 28cNF do not lack transcription of genes in these regions, suggesting that these deletions are likely an artifact. This analysis demonstrates that the sex of each cell line was as expected, and that the immortalization process did result in large copy number alterations in the cNF cell lines. (PDF) [file pone.0340183.s005.pdf]
