# Supplementary material for: Immortalization and characterization of Schwann cell lines derived from NF1-associated cutaneous neurofibromas
Source: PLoS One. 2026 Jan 21;21(1):e0340183. doi: 10.1371/journal.pone.0340183 (PMC12822933; doi:10.1371/journal.pone.0340183)
Supplement: S5 Table — (PDF) [file pone.0340183.s017.pdf]

Cell Line	Variant Classification	Variant Type	Genetic Change	Protein Change	dbSNP ID
28cNF	Nonsense_Mutation	SNP	c.3233C>G	p.S1078*	rs2067122696
i28cNF	Nonsense_Mutation	SNP	c.3158C>G	p.S1053*	rs1597717610
i28cNF	Nonsense_Mutation	SNP	c.3233C>G	p.S1078*	rs2067122696
cNF00.10a	Nonsense_Mutation	SNP	c.5861C>A	p.S1954*	
icNF00.10a	Nonsense_Mutation	SNP	c.5861C>A	p.S1954*	
cNF04.9a	Splice_Site	SNP	c.5268+2T>G	p.X1756_splice	rs1555533416
cNF04.9a	Frame_Shift_Del	DEL	c.7391_7407del	p.T2464Kfs*13	novel
icNF04.9a	Splice_Site	SNP	c.5268+2T>G	p.X1756_splice	rs1555533416
icNF04.9a	Nonsense_Mutation	SNP	c.5503C>T	p.Q1835*	
icNF04.9a	Frame_Shift_Del	DEL	c.7391_7407del	p.T2464Kfs*13	novel
cNF97.2a	Frame_Shift_Del	DEL	c.233del	p.N78Ifs*7	rs1438566555
cNF97.2a	Frame_Shift_Del	DEL	c.1929del	p.M643Ifs*45	rs1567846742
icNF97.2a	Frame_Shift_Del	DEL	c.233del	p.N78Ifs*7	rs1438566555
icNF97.2a	Frame_Shift_Del	DEL	c.1929del	p.M643Ifs*45	rs1567846742
cNF97.2b	Frame_Shift_Del	DEL	c.233del	p.N78Ifs*7	rs1438566555
cNF97.2b	Splice_Site	DEL	c.1392+2_1392+3del	p.X464_splice	novel
icNF97.2b	Frame_Shift_Del	DEL	c.233del	p.N78Ifs*7	rs1438566555
icNF97.2b	Splice_Site	DEL	c.1392+2_1392+3del	p.X464_splice	novel
cNF98.4c	Splice_Site	SNP	c.6704+1G>T	p.X2235_splice	rs1060500376
icNF98.4c	Splice_Site	SNP	c.6704+1G>T	p.X2235_splice	rs1060500376
cNF98.4d	Frame_Shift_Del	DEL	c.6316del	p.V2106Lfs*5	
cNF98.4d	Splice_Site	SNP	c.6704+1G>T	p.X2235_splice	rs1060500376
icNF98.4d	Frame_Shift_Del	DEL	c.6316del	p.V2106Lfs*5	
icNF98.4d	Splice_Site	SNP	c.6704+1G>T	p.X2235_splice	rs1060500376

S5 Table. NF1 mutations detected by targeted sequencing are recapitulated using whole-genome sequencing. Orange indicates primary cell cultures, while green indicates immortalized cell lines.
