# Supplementary material for: Immortalization and characterization of Schwann cell lines derived from NF1-associated cutaneous neurofibromas
Source: PLoS One. 2026 Jan 21;21(1):e0340183. doi: 10.1371/journal.pone.0340183 (PMC12822933; doi:10.1371/journal.pone.0340183)
Supplement: S6 Table — (PDF) [file pone.0340183.s018.pdf]

Primary cell line	Immortalized cell line	Jaccard index
28cNF	i28cNF	0.722
cNF00.10a	icNF00.10a	0.714
cNF04.9a	icNF04.9a	0.715
cNF97.2a	icNF97.2a	0.679
cNF97.2b	icNF97.2b	0.717
cNF98.4c	icNF98.4c	0.720
cNF98.4d	icNF98.4d	0.712

S6 Table. Jaccard indices of the WGS variant profiles identified in the paired primary and immortalized cell lines.
