# Supplementary material for: Immortalization and characterization of Schwann cell lines derived from NF1-associated cutaneous neurofibromas
Source: PLoS One. 2026 Jan 21;21(1):e0340183. doi: 10.1371/journal.pone.0340183 (PMC12822933; doi:10.1371/journal.pone.0340183)

S19 Figure. **Euclidean distances between primary cultures and immortalized cell lines based on complete gene expression profiles.** Hierarchical clustering of the Euclidean distances show that the transcriptome profiles of primary cultures from the same patient and their immortalized cell lines were more similar (lower distance) to each other than to that of primary cultures and immortalized cell lines from other patients or other tumor types (e.g. plexiform neurofibromas: hTERT_NF1_ipNF95.11b_C and hTERT_NF1_ipNF95.6).

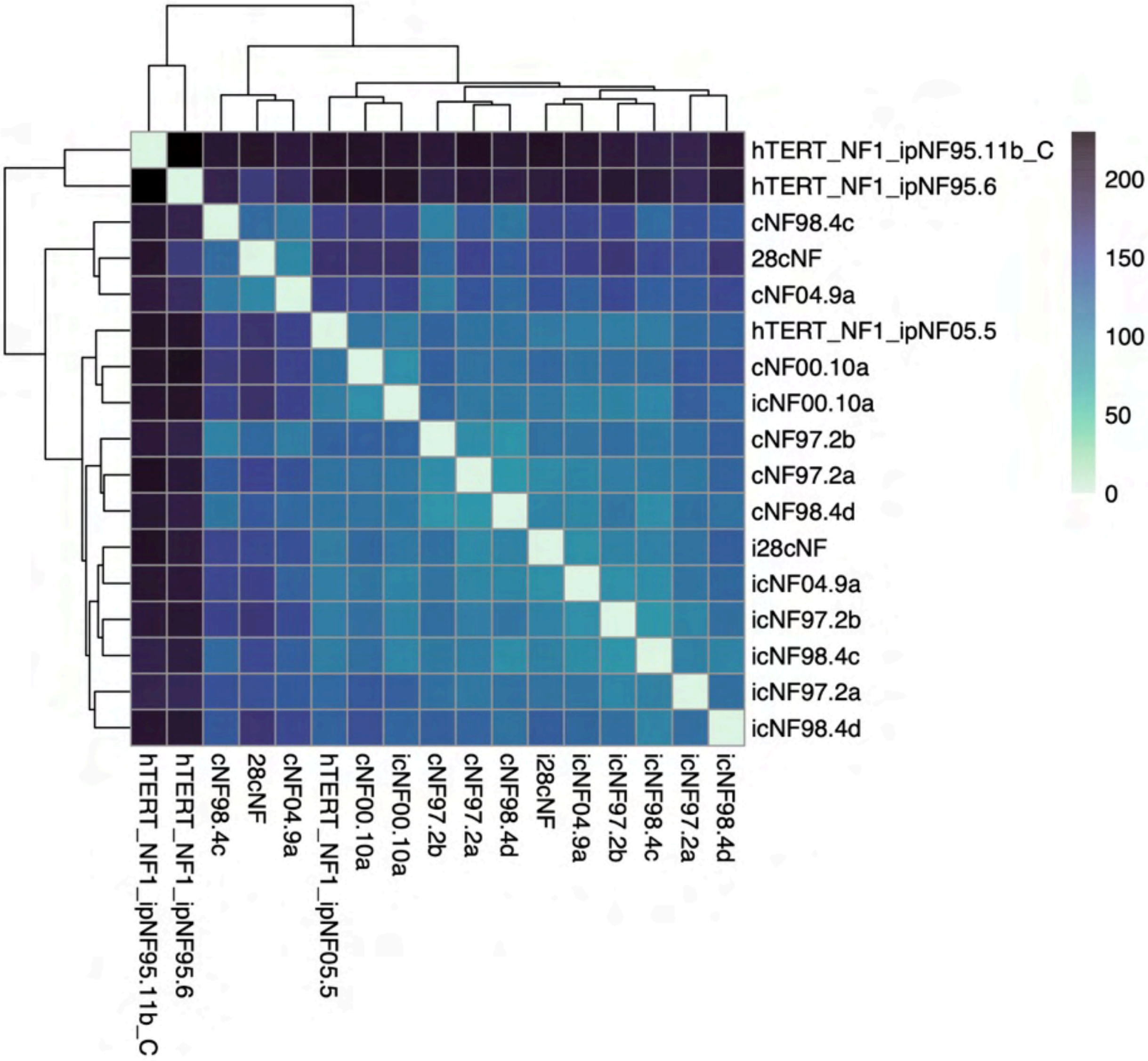

Supplement: S19 Fig — Hierarchical clustering of the Euclidean distances show that the transcriptome profiles of primary cultures from the same patient and their immortalized cell lines were more similar (lower distance) to each other than to that of primary cultures and immortalized cell lines from other patients or other tumor types (e.g., plexiform neurofibromas: hTERT_NF1_ipNF95.11b_C and hTERT_NF1_ipNF95.6). (PDF) [file pone.0340183.s006.pdf]
